# TridentSynth: a webtool for the retrosynthesis of molecules using chimeric type I polyketide synthases and chemoenzymatic pathways

**DOI:** 10.1093/nar/gkag471

**Published:** 2026-05-11

**Authors:** Yash Chainani, Margaret Guilarte-Silva, Kenna Roberts, Stefan Pate, Geoffrey Bonnanzio, Keith E J Tyo, Aindrila Mukhopadhyay, Jay D Keasling, Hector Garcia Martin, Linda J Broadbelt, Tyler W H Backman

**Affiliations:** Department of Chemical and Biological Engineering, Northwestern University, Evanston, IL 60208, United States; Center for Synthetic Biology, Evanston, IL 60208, United States; Joint BioEnergy Institute, Emeryville, CA 94608, United States; Biological Systems and Engineering Division, Lawrence Berkeley National Laboratory, Berkeley, CA 94720, United States; Department of Chemical and Biological Engineering, Northwestern University, Evanston, IL 60208, United States; Center for Synthetic Biology, Evanston, IL 60208, United States; Department of Chemical and Biological Engineering, Northwestern University, Evanston, IL 60208, United States; Center for Synthetic Biology, Evanston, IL 60208, United States; Joint BioEnergy Institute, Emeryville, CA 94608, United States; Biological Systems and Engineering Division, Lawrence Berkeley National Laboratory, Berkeley, CA 94720, United States; Department of Chemical and Biological Engineering, Northwestern University, Evanston, IL 60208, United States; Center for Synthetic Biology, Evanston, IL 60208, United States; Department of Chemical and Biological Engineering, Northwestern University, Evanston, IL 60208, United States; Center for Synthetic Biology, Evanston, IL 60208, United States; Department of Chemical and Biological Engineering, Northwestern University, Evanston, IL 60208, United States; Center for Synthetic Biology, Evanston, IL 60208, United States; Joint BioEnergy Institute, Emeryville, CA 94608, United States; Biological Systems and Engineering Division, Lawrence Berkeley National Laboratory, Berkeley, CA 94720, United States; Joint BioEnergy Institute, Emeryville, CA 94608, United States; Biological Systems and Engineering Division, Lawrence Berkeley National Laboratory, Berkeley, CA 94720, United States; QB3, University of California, Berkeley, CA 94720, United States; Department of Chemical and Biomolecular Engineering, University of California, Berkeley, CA 94720, United States; Department of Bioengineering, University of California, Berkeley, CA 94720, United States; Center for Biosustainability, Danish Technical University, Lyngby 2800, Denmark; Joint BioEnergy Institute, Emeryville, CA 94608, United States; Biological Systems and Engineering Division, Lawrence Berkeley National Laboratory, Berkeley, CA 94720, United States; BCAM, Basque Center for Applied Mathematics, 48009 Bilbao, Spain; DOE Agile BioFoundry, Emeryville, CA 94608, United States; Department of Chemical and Biological Engineering, Northwestern University, Evanston, IL 60208, United States; Center for Synthetic Biology, Evanston, IL 60208, United States; Joint BioEnergy Institute, Emeryville, CA 94608, United States; Biological Systems and Engineering Division, Lawrence Berkeley National Laboratory, Berkeley, CA 94720, United States; Joint BioEnergy Institute, Emeryville, CA 94608, United States; Biological Systems and Engineering Division, Lawrence Berkeley National Laboratory, Berkeley, CA 94720, United States

## Abstract

The design of pathways to synthesize valuable molecules remains a central challenge in chemistry and biotechnology. Several computational retrosynthesis tools have been developed to address this problem, but their scope is often confined only to reactions in either synthetic organic chemistry or monofunctional enzymatic chemistry. We present TridentSynth, a web-based retrosynthesis tool (https://tridentsynth.lbl.gov) to scale synthesis planning up to three different routes by also incorporating multifunctional Type I polyketide synthase (PKS) enzymes into our reaction toolkit along with organic chemistry and monofunctional enzymes. Unlike monofunctional enzymes that catalyze single transformations, PKSs function as molecular assembly lines that catalyze multiple carbon-carbon bond formation reactions between acyl-coenzyme A substrates to construct elongated carbon scaffolds. PKSs follow a modular, programmable logic that allows them to be reconfigured to make new molecules in a predictable way. These scaffolds can then be chemoenzymatically modified to eventually access a wider array of molecular targets than would be possible with just synthetic chemistry or monofunctional enzymes alone, in a manner that mimics the evolved biosynthesis routes of many useful natural products. TridentSynth assists synthetic biologists by suggesting routes to synthesize a desired molecule through an intuitive web interface that requires no local installation or programming expertise.

## Graphical Abstract

**Graphical Abstract:**
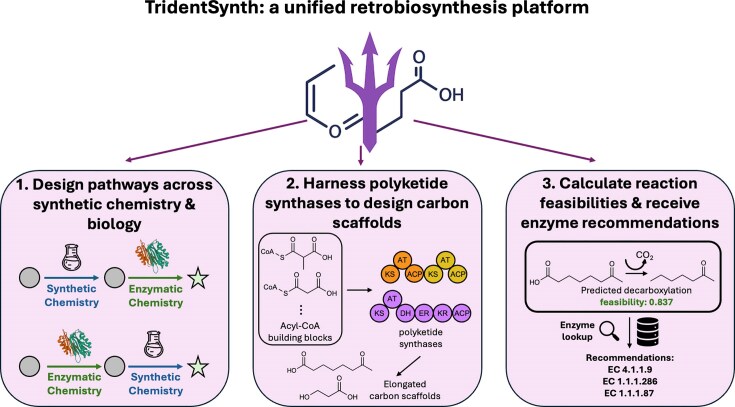

## Introduction

Chemistry and biology offer complementary advantages for the multistep synthesis of valuable molecules [1–7], as exemplified by monofunctional enzymes and chemically catalyzed reactions. Monofunctional enzymes in biology catalyze single reactions, such as alcohol dehydrogenases or monooxygenases, that excel at regioselectively and stereoselectively introducing precise modifications to select functional groups on a given substrate’s carbon backbone [8, 9]. Most enzymes, however, can only function within a narrow range of physiologically relevant temperatures, pressures, and pH values and will typically denature when forced outside of this range [9]. Chemically catalyzed reactions, however, are not confined to such limited conditions and are consequently able to perform a much wider suite of functional group transformations than biological reactions [10–12]. By merging the breadth of transformations available through chemistry with the precision and selectivity of enzymatic catalysis, hybridized chemoenzymatic syntheses can ultimately access a much larger chemical space of products than would be possible using either chemistry or monofunctional enzymes in isolation [5, 13, 14]. This complementary synthesis paradigm has been demonstrated experimentally in the chemoenzymatic synthesis of papaverine [15] and methacrylic acid [16], among many other complex and value-added molecules [17–19].

In addition to monofunctional enzymes and synthetic chemistry, a third and relatively under-utilized synthesis route provides access to an even wider chemical space of molecular targets: multifunctional enzymes. In contrast to monofunctional enzymes that catalyze only a single reaction, multifunctional enzymes catalyze multiple reactions. Among these systems, Type I polyketide synthases (PKSs) are particularly notable owing to their modular nature [20–26]: type I PKSs can be conceptualized as an assembly line comprising smaller enzymatic domains operating in concert to iteratively condense and modify simple acyl-coenzyme A (acyl-CoA) substrates, such as malonyl-CoA or methylmalonyl-CoA, into a range of elongated and functionally diverse scaffolds [20–26]. In nature, PKSs are genomically encoded within large biosynthetic gene clusters present within many bacterial genomes and are responsible for the biosynthesis of many structurally complex and therapeutically relevant natural products [27–30]. This unique ability of PKSs to repeatedly catalyze carbon-carbon bond formation reactions makes them an attractive tool for the bioproduction of low to mid-molecular weight molecules (<1000 Da), particularly those that require extended carbon chain backbones. Recent work has also shown that PKSs can be successfully used in conjunction with monofunctional enzymes to synthesize a wide array of chemicals [31–33]. Indeed, we have previously released a retrosynthesis tool, BioPKS Pipeline [34], that integrates the design of chimeric type I PKSs with monofunctional enzymes. BioPKS Pipeline is in turn built upon two different pathway design tools: RetroTide, which designs chimeric type I PKSs, and DORAnet, which designs novel biosynthetic pathways using monofunctional enzymes [35].

Retrosynthesis tools can greatly accelerate the design of novel pathways by combining and permuting both known and predicted reactions to generate large *in silico* reaction networks [36–50]. These networks can then be traversed to extract subgraphs of pathways connecting precursors of interest to the desired target molecule. Without such pathway design tools, synthesis planning would be prohibitively time-consuming, requiring chemists or biologists to manually survey the literature for all possible known reactions while simultaneously hypothesizing where any novel transformations may be possible. These tasks become exponentially harder as pathways get longer and comprise more reaction steps. Despite their utility, many existing retrosynthesis platforms remain difficult for experimental practitioners to adopt, as they are often distributed as complex software packages with extensive dependencies that require substantial computational expertise to install, configure, and maintain. As a result, the practical use of these tools in experimental workflow planning remains limited [51, 52].

**Figure 1.**
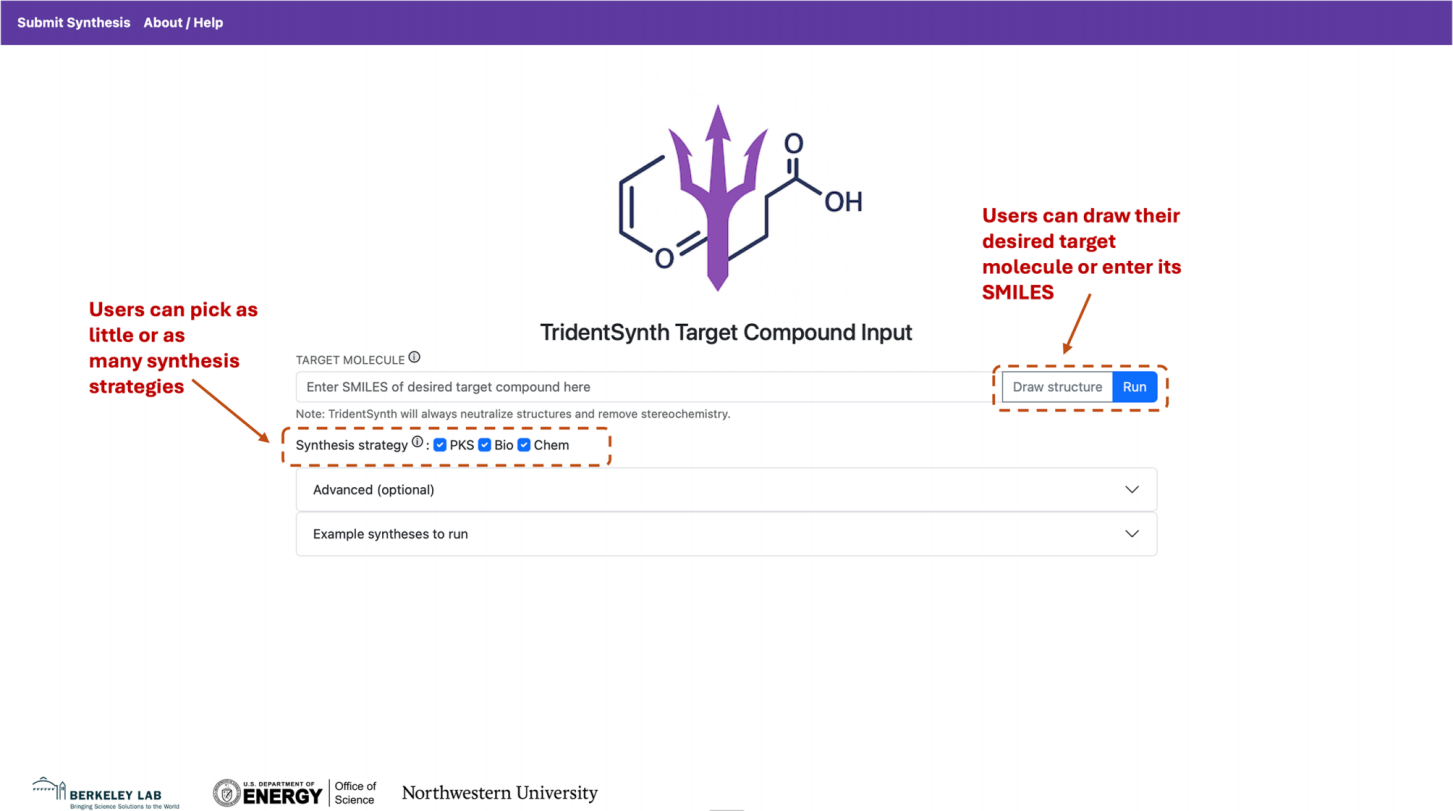
TridentSynth webserver landing page and target submission interface. The TridentSynth web interface allows users to submit a target molecule as a SMILES string or via an interactive structure editor. Users can select one or more synthesis strategies: PKS-based biosynthesis or non-PKS enzymatic synthesis, and/or synthetic chemistry. Advanced options and example targets are also available to help guide users in determining the types of PKS acyl-CoA substrates they would like to work with and/or the number of chemoenzymatic tailoring reactions.

Here, we present a merger of three synthesis modalities (PKS, monofunctional enzymes, chemical reactions) into a single, unified platform, TridentSynth, which substantially expands the chemical space accessible to computational retrosynthesis. The TridentSynth webtool was designed to be user-friendly (Fig. 1) and accessible to enable synthetic biologists and chemists to rapidly explore and evaluate synthesis pathways that can be directly implemented in the laboratory, without requiring local software installation or programming expertise. The TridentSynth webtool has been deployed as a web-based application, with a lightweight web frontend and a Django/Python-based backend that orchestrates synthesis planning. Task queueing and load balancing are handled using Celery and Redis, allowing our server to efficiently manage multiple user-submitted pathway design jobs. From the landing page (Fig. 1), users specify a target molecule of interest and select one or more synthesis modalities: PKS-based, enzymatic, chemical, or hybrid to guide pathway generation. When a PKS-based synthesis route is selected, TridentSynth operates in the forward direction, proposing candidate polyketide scaffolds constructed from simple acyl-CoA building blocks that maximize chemical similarity to the target molecule. These biosynthetically accessible scaffolds serve as starting points for downstream transformations and can be further modified through monofunctional enzymatic reactions, synthetic organic reactions, or combined chemoenzymatic steps to reach the final target. Alternatively, users may choose a non-PKS-based synthesis, in which case TridentSynth performs backward pathway search starting from the input target molecule and searches for high-flux metabolites and other readily available precursors. By supporting both forward PKS-driven scaffold construction and backward retrosynthetic analysis within a single interface, our TridentSynth webtool enables users to seamlessly explore complementary synthesis strategies and compare the resulting candidate pathways. We have made TridentSynth free and open to all users and there is no login requirement. It can be accessed at https://tridentsynth.lbl.gov.

## Materials and methods

### Architecture of the TridentSynth webtool

**Figure 2.**
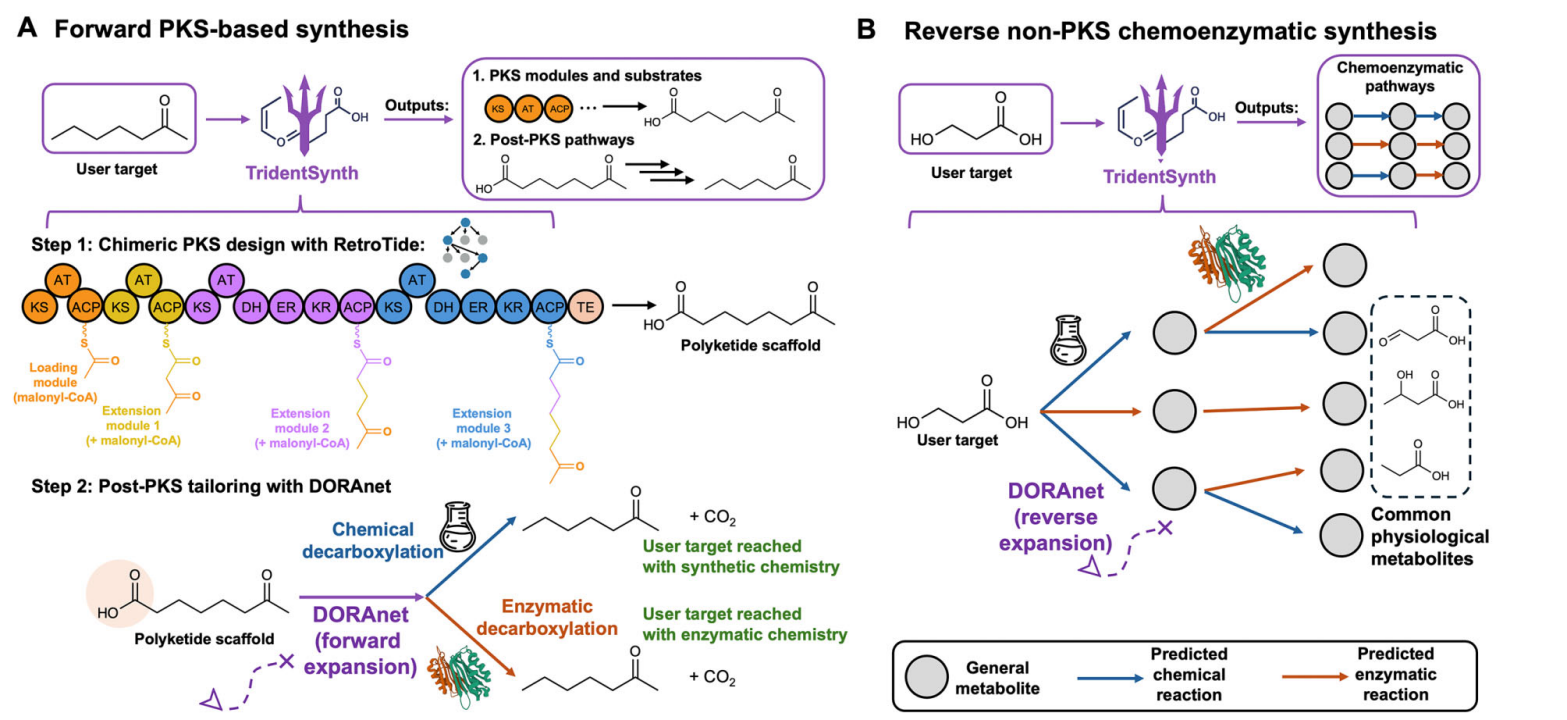
TridentSynth performs both PKS-based forward synthesis and non-PKS retrosynthesis. **(A)** If a PKS-based synthesis route is chosen, TridentSynth takes a target molecule and first designs a chimeric PKS using RetroTide to construct a candidate polyketide carbon scaffold. Here, each color denotes a distinct PKS module, and carbon atoms in the growing polyketide chain are colored according to the module responsible for their incorporation. Following scaffold assembly, DORAnet performs forward expansion from the polyketide intermediate, applying enzymatic and/or chemical reaction rules to generate post-PKS tailoring steps that lead to the target molecule. **(B)** When a non-PKS route is selected, TridentSynth invokes only DORAnet and applies it in the retrosynthetic direction by working backwards from the target molecule using chemical and/ or enzymatic reaction rules until common metabolites are reached.

The TridentSynth webtool is deployed as a Django-based web application with an interactive frontend that allows users to specify their target molecule either by entering a SMILES string or by drawing a molecular structure directly. In addition to defining the target, users can configure a range of synthesis parameters, including the synthesis routes to explore, the maximum number of reaction steps, the PKS release mechanism, and the choice of starter and extender substrates for a PKS-based synthesis. Upon submission of these synthesis parameters, the frontend issues an asynchronous javascript and XML (AJAX) POST request to the backend, which first sanitizes the SMILES string of the input target molecule and checks for its chemical validity using the cheminformatics package RDKit. All stereochemistry information is also removed from this input SMILES string. If any electronic charges are present, this SMILES string is converted into its neural form. Invalid or malformed SMILES strings are rejected, and the TridentSynth webtool sends an error message back to the frontend, prompting the user to re-examine their SMILES input. For valid submissions, the backend packages the sanitized input and user-defined parameters into a synthesis job, which executes the full retrosynthesis workflow with the specified hyperparameters. TridentSynth builds on our previously released BioPKS Pipeline (available at https://github.com/JBEI/BioPKS-Pipeline/tree/main​​), which integrated the design of multifunctional chimeric type I PKSs with monofunctional enzymatic modifications. TridentSynth extends this framework by additionally incorporating synthetic organic chemistry reactions, enabling the design of fully chemoenzymatic pathways. RetroTide designs chimeric PKSs, and DORAnet designs non-PKS chemoenzymatic pathways (Fig. 2). The architecture of both RetroTide and DORAnet as well as their use of reaction networks and templates for the design of synthesis pathways has been detailed extensively in our previous publications [35, 53].

Once synthesis jobs have been created, they are dispatched asynchronously using Celery and placed into a distributed task queue, with Redis serving as both the message broker and the results backend. This architecture helps to decouple incoming web requests from their execution, thereby allowing longer-running synthesis jobs to be executed sequentially from a queue by dedicated worker processes without overloading the TridentSynth webserver. Each job’s metadata and execution status are stored in Redis and exposed through a REpresentational State Transfer application programming interface, which the frontend periodically queries to provide users with updates on the status of their job. While a job is running, users will also be given a webpage link that they can bookmark and to which they can return.

**Figure 3.**
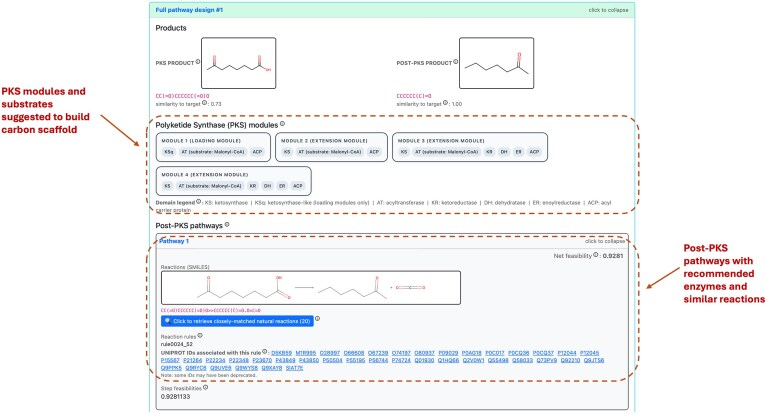
TridentSynth outputs pathway designs with rich visual annotations. The results interface shown here summarizes the PKS-based forward synthesis of 2-heptanone, displaying predicted intermediates, PKS modules, and post-PKS transformations in a unified visual layout. The PKS-derived scaffold (7-oxooctanoic acid) and final product are shown with similarity-to-target scores, alongside the corresponding chimeric PKS design specifying the acyl-CoA substrates and catalytic domains required to build this scaffold. Post-PKS chemoenzymatic tailoring steps are presented as annotated reaction panels, including reaction SMILES, associated reaction rules, and linked UniProt identifiers for candidate enzymes. For enzymatic reactions, TridentSynth reports machine-learning derived reaction feasibility scores at both the step and pathway levels, enabling rapid visual assessment and prioritization of predicted biosynthetic routes.

After a synthesis job has been completed, the TridentSynth webtool navigates away from the waiting page and brings the user to a results page that starts with a job summary panel displaying various metadata, such as Task ID, target SMILES, and a rendered image of the target structure (Fig. 3). Below this summary, the results page displays a series of pathway cards. Each card is color-coded (green for when pathways were found to the full desired target and yellow when pathways reached only a chemically similar target) and collapsible, so that users can scan the list and expand only pathway designs that they wish to investigate further. Additional information on the architecture of our webtool and the sequence of operations that occurs once a synthesis job is submitted on the frontend can also be found in the Supplementary Information section (Supplementary Fig. S1 and Supplementary Fig. S2).

### Ranking post-PKS pathways and enzyme recommendations

Chemoenzymatic synthesis routes generated by TridentSynth are ranked using a combination of rule-based reaction annotations and machine-learning–derived reaction feasibility scoring [54]. For each chemoenzymatic transformation proposed by DORAnet, TridentSynth records the underlying reaction rule used to generate the transformation. For enzymatic transformations, TridentSynth also displays the associated set of UniProt identifiers corresponding to enzymes that may catalyze the predicted reaction. These enzyme associations are drawn from our previously published reaction rule databases [55, 56], which link chemical transformations to experimentally observed enzymatic reactions. This rule-based framework ensures that all proposed enzymatic steps can be traced back to known biochemical reactions [55, 56]. Given that reaction networks grow exponentially with each generation, this reaction annotation and prioritization step is essential for managing the large number of candidate chemoenzymatic pathways that can be generated from a single starting molecule.

TridentSynth further ranks pathways that involve monofunctional enzymatic reactions by assessing the feasibility of each enzymatic transformation using our previously published DORA-XGB supervised reaction classification model [54]. For each reaction step, the model outputs a feasibility score ranging from 0.0 to 1.0, reflecting the likelihood that the proposed substrate–product pairing corresponds to a biochemically plausible transformation, with values closer to 1.0 indicating higher feasibility. For multistep pathways, individual reaction scores are combined by taking their product to yield an overall pathway feasibility score, enabling direct comparison across alternative pathways. These feasibility scores are reported by TridentSynth alongside the corresponding reaction rules [55, 56] and UniProt identifiers [57] in the results interface, allowing users to evaluate both reaction-level confidence and cumulative pathway plausibility when prioritizing designs for downstream analysis or experimental implementation. At present, our feasibility-based ranking is limited only to monofunctional enzymatic reactions [54]; however, we aim to extend analogous scoring frameworks to PKS-based designs and synthetic chemistry reactions in future versions of the platform.

### Extracting a list of common biological metabolites as starting points for synthesis

All biological metabolites and their corresponding source organisms were extracted from a previously downloaded version of the MetaCyc database [58, 59] (V24.0, released 2020) that we had accessed for a different publication [55]. We extracted intersection of metabolite compounds from representative metabolomes of the most common host organisms used in metabolic engineering: *Escherichia coli, Streptomyces cerevisiae, Yarrowia lipolytica, Rhodococcus jostii, Bacillus subtilis, Pseudomonas putida, Acinetobacter baylyi, Corynebacterium glutamicum*, and *Mycobacterium tuberculosis*. This resulted in 334 distinct metabolites common to all these organisms, from which their corresponding SMILES strings were stored in neutral, canonical form, and without any stereochemistry.

If the user selects a non-PKS synthesis route, encompassing monofunctional enzymatic transformations, purely chemical reactions, or hybrid chemoenzymatic strategies, DORAnet performs a retrosynthetic expansion of the target molecule for the user-specified number of steps and then checks whether any of the resulting precursors appear in this curated set of 334 common metabolites. Because these metabolites are typically present at high intracellular concentrations, and present in nearly all bioproduction chassis microorganisms, they serve as practical and biologically realistic starting points for pathway design. Providing this predefined precursor set also removes the need for users to manually identify suitable biological starting compounds themselves.

### Reaction similarity search via reaction center-based Morgan fingerprints

To quantify the similarity between a predicted enzymatic reaction and known natural reactions, we developed a Reaction Center Morgan Fingerprint (RCMFP) based on Extended Connectivity Fingerprints (ECFP) [60]. We customized the RDKit Morgan fingerprint implementation to encode reaction-specific information. Each atom is initially represented by standard molecular descriptors, such as degree, total valence, number of hydrogen neighbors, atomic number, formal charge, ring membership, aromaticity, and number of heteroatom neighbors as well as the minimum topological distance to each reaction center atom. Subgraphs up to a radius of 2 centered on each atom are then hashed to produce a 2048-bit binary vector for each side of the reaction. When comparing two reactions, we permute one reaction’s direction and take the higher of the two Tanimoto similarity scores. Additionally, we verify that the reaction centers of the two reactions are equivalent; if they are not, the similarity score is set to zero. The full RCMFP implementation is available at https://github.com/stefanpate/ergochemics.

We applied this fingerprint to a database of 55 228 known enzyme-catalyzed reactions, which was curated from all atom-mapped versions of the BRENDA [61], KEGG [62–65], and MetaCyc [58] databases released by the EnzymeMap database [66] (downloaded from https://github.com/hesther/enzymemap and https://zenodo.org/records/8254726). Out of these 55 228 reported enzymatic reactions, 54 063 (97.9%) were successfully fingerprinted. Fingerprints were pre-computed offline and stored as a NumPy compressed archive (.npz file) to enable rapid retrieval at query time. To ensure chemical relevance, the similarity search is constrained to reactions belonging to the same generalized rule family [55] as the query reaction, i.e. reactions that share the same reaction center transformation. If no reactions in the database match the query rule family, the search falls back to the full database. For each predicted enzymatic step, TridentSynth returns the 20 most similar known reactions along with their EC classifications and hyperlinks to relevant BRENDA [61], KEGG [62–65], and MetaCyc [58] pages, providing users with a ranked set of candidate enzymes for experimental follow-up.

### Checking for valid inputs and prohibited chemicals

In order to warn users if they input a controlled substance, we have curated a list of 652 banned chemicals from the open chemical weapons convention (https://www.opcw.org). The SMILES strings of these 652 chemicals are stored in their neutral, canonical form, and without any stereochemistry. If a chemical from this list is entered as input, TridentSynth displays a warning alerting the user that the compound may be a controlled or prohibited substance. Users may still proceed with the synthesis if they have a legitimate research purpose, as many of these compounds remain under active investigation.

## Results

### Design of merged PKS and post-PKS pathways

The TridentSynth webtool enables users to design synthesis pathways to target molecules of interest using either a PKS-based or a non-PKS based synthesis route. Users can specify the synthesis modalities they wish to use directly on the landing page of our webtool. The landing page also includes an optional advanced settings panel that allows users to configure additional synthesis parameters, including the number of post-PKS biological and chemical synthesis tailoring steps (ranging from 1 to 3, with a default of 1 for each), the PKS chain release mechanism (either thiolysis or cyclization, with the default set to thiolysis), and the choice of acyl-CoA starter and extender substrates (defaults set to malonyl- and methylmalonyl-CoA). These defaults are applied automatically if the panel is not expanded, and were intentionally selected to balance pathway feasibility with computation time because with each step, reaction networks grow exponentially, thereby leading to significantly longer runtimes.

If a PKS-based synthesis is chosen, then upon receiving the target SMILES string from the frontend, the TridentSynth webtool first calls on RetroTide [35], our previously released PKS design software, in the backend to design the carbon backbone of this molecule using only PKSs (Fig. 2A). In order to build this carbon backbone, RetroTide combines and permutes different PKS enzymatic domains with various acyl-CoA substrates to build a diverse range of polyketide scaffolds. Users have the option on the frontend to select which acyl-CoA starter and extender units should be used to construct these scaffolds. While malonyl-CoA and methylmalonyl-CoA are the most common naturally occurring substrates and will be selected as the default, we also allow users to select from a library of both rarer and unnatural substrates, such as cinnamoyl-CoA [67] and allylmalonyl-CoA [68, 69], which may be helpful for synthesizing more structurally complex and functionally diverse targets. On the backend, the outputs from RetroTide are (i) a list of PKS designs, each in turn comprising a list of domains and acyl-CoA substrates, and (ii) a list of corresponding scaffolds. These PKS designs are ranked in descending order of the chemical similarity between their corresponding scaffold and the target molecule. In the event that the top-ranked PKS design can already reach the input target, i.e. a maximum common substructure-based Tanimoto similarity score of 1.0 is obtained, then the webtool displays a message indicating that the target has already been reached and does not perform any additional post-PKS chemoenzymatic modifications, even if these were initially requested. PKS designs represent a series of reactions and domains, and do not guide the user in the selection of specific DNA parts and junctions that might catalyze these steps, which is left up to the user.

If the top-ranked PKS design does not lead to the target, however, then the requested post-PKS modifications are performed by DORAnet [35], our previously released chemoenzymatic pathway design software, on the backend (Fig. 2A). These chemoenzymatic tailoring reactions are performed sequentially, with synthetic organic chemistry reactions applied first, followed by monofunctional enzymatic reactions. Users may request either type of modification individually or in combination. For enzymatic tailoring reactions, the TridentSynth webtool also provides reaction feasibility scores output by our previously published machine learning model [54] as well as UniProt identifiers [57] of potentially promiscuous enzymes to catalyze predicted reactions. These reaction feasibility scores range from 0.0 to 1.0, with higher scores indicating more feasible reactions [54]. The enzyme recommendations provided are derived from our previously published set of reaction rules, which encode experimentally supported enzymatic transformations curated from the literature and biochemical databases, with each rule linked to corresponding UniProt identifiers [57] for enzymes reported to catalyze the transformation [55].

If a PKS-based synthesis route is not chosen, i.e. only chemical, enzymatic, or chemoenzymatic transformations are requested by the user, then the TridentSynth webtool backend does not invoke RetroTide and relies only on DORAnet instead. Further, rather than building towards the target from simple acyl-CoA building blocks in the forward direction, the target is retrosynthetically expanded upon in the reverse direction to generate a list of upstream precursors (Fig. 2B). Although many of these upstream precursors may be novel compounds, we screen them against a curated set of 334 common biological metabolites drawn from the MetaCyc database [58]. These are present across most common microbial bioproduction chassis organisms, and therefore represent viable starting points for synthesis. Pathways that terminate in these known, accessible metabolites are retained for further evaluation (Fig. 2B).

### Interactive visualization of predicted synthesis pathways

The TridentSynth webtool presents predicted synthesis routes using an integrated, interactive visualization that consolidates molecular structures, PKS modules, and reaction-level annotations within a single interface (Fig. 3). For each candidate pathway, the results page summarizes both the modular PKS logic to build the target’s carbon scaffold and the chemoenzymatic modifications required, enabling rapid interpretation and comparison of various routes.

Consider the example of planning synthesis of 2-heptanone using PKSs and post-PKS monofunctional enzymatic reactions. Within the first full pathway design, labelled “Full pathway design #1” below (Fig. 3), our interface displays the molecular structures of the predicted PKS intermediate, 7-oxooctanoic acid, and the final product, 2-heptanone, reached after post-PKS reactions. Both structures are rendered with the cheminformatics package RDKit and have been annotated with their chemical similarity scores with respect to the 2-heptanone target. These similarity scores allow users to evaluate how closely the PKS-generated scaffold matches the target molecule prior to downstream modification. Underneath these molecular structures, our interface prints out the chimeric type I PKS design proposed to construct the 7-oxooctanoic acid intermediate. In this predicted design, the PKS features one loading module followed by three extension modules, with all modules selecting malonyl-CoA as their substrate. The interface also displays the enzymatic domains present within each module. In the example shown, the final two modules, i.e. extension module 3 and extension module 4, are fully reducing since they comprise ketoreductase (KR), dehydratase (DH), and enoylreductases (ER) domains in their design. These fully reducing loops help create the saturated carbon backbone required for 2-heptanone. Across all modules, the presence of the ketosynthase (KS) domain, which is responsible for catalyzing Claisen condensation reactions between the previous module’s polyketide substrate and a new extender substrate, is implicitly assumed and not visually shown.

Post-PKS chemoenzymatic modifications are presented as expandable pathway panels that detail the tailoring steps applied to the PKS-derived intermediate. In this example, the 7-oxooctanoic acid scaffold undergoes a single DORAnet-predicted enzymatic decarboxylation reaction, converting it to the final target molecule, 2-heptanone. Each post-PKS reaction is displayed with its corresponding reaction image, reaction SMILES, the reaction rule used to generate the transformation, and a list of linked UniProt identifiers representing candidate enzymes reported to catalyze the reaction. Hyperlinks to external UniProt entries are also provided to allow users to rapidly access relevant information for the proposed enzymes, such as their primary amino acid sequence or three-dimensional structure. For non-PKS enzymatic steps, TridentSynth additionally reports machine-learning-derived feasibility scores at both the individual reaction and overall pathway levels (Fig. 3), enabling prioritization of biochemically plausible routes.

Together, this interactive visualization framework provides a unified and navigable view of predicted intermediates, PKS module architectures, enzymatic transformations, and feasibility metrics, supporting efficient exploration, comparison, and experimental planning of candidate synthesis pathways.

When a PKS-based synthesis route is not selected, TridentSynth operates exclusively in a non-PKS retrosynthetic mode, using DORAnet to identify enzymatic and/or chemical pathways that may connect the target molecule to at least one metabolite in a curated set of 334 common biological metabolites. In this mode, synthesis planning proceeds in the reverse direction, starting from the target compound and iteratively expanding upstream through enzyme-catalyzed and/or synthetic chemistry reactions. PKS module design is omitted entirely, and pathway generation instead focuses solely on identifying transformations that link the target to metabolites that are already abundant and accessible within common cellular environments.

**Figure 4.**
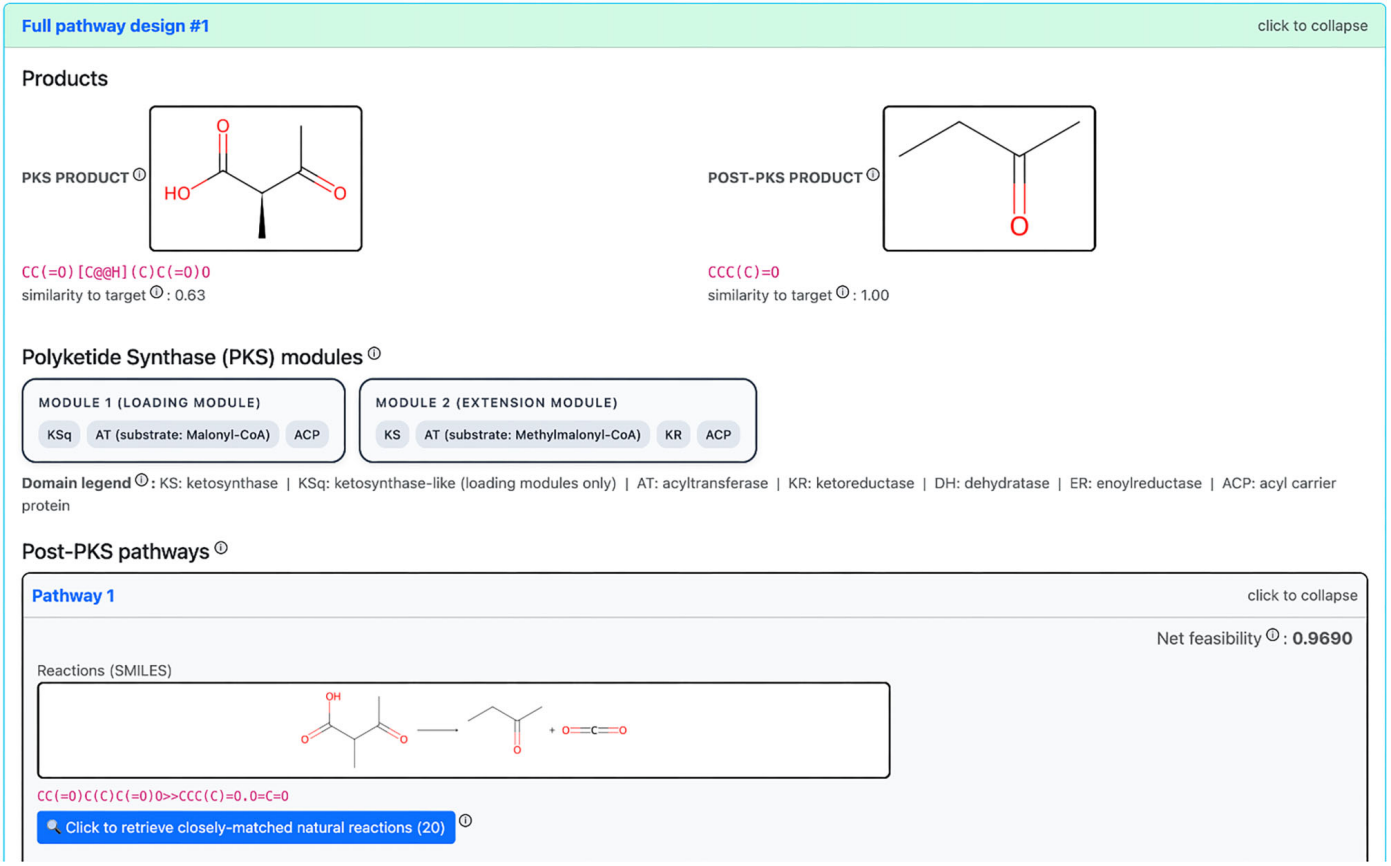
Proposed biosynthetic pathway to 2-butanone from TridentSynth via a chimeric PKS and a single post-PKS enzymatic step. The suggested chimeric PKS design comprises one loading module that uses malonyl-CoA as a starter unit and an extension module that uses methylmalonyl-CoA as an extender unit. This results in 2-methyl-3-oxobutanoic acid as a polyketide product intermediate upon thiolysis. A single post-PKS β-keto acid decarboxylation then converts this intermediate to the final 2-butanone product, with a DORA-XGB feasibility score of 0.969.

Figure 4 illustrates another combined PKS and enzymatic tailoring workflow using 2-butanone as the target molecule. In this example, TridentSynth invokes RetroTide to design a two-module chimeric type I PKS comprising a malonyl-CoA loading module and a single extension module utilizing methylmalonyl-CoA. Upon thiolysis release, this PKS produces 2-methyl-3-oxobutanoic acid, a β-keto acid intermediate. TridentSynth then applies DORAnet to identify a single post-PKS decarboxylation step that converts this intermediate to 2-butanone with release of CO₂. Our DORA-XGB feasibility classifier assigns this reaction a score of 0.969, and the enzyme retrieval module identifies methyloxaloacetate decarboxylase (EC 4.1.1.112) as a closely matched natural enzyme whose native substrate, methyloxaloacetate, shares the same β-keto acid core and methyl branch as the proposed intermediate, differing only in a terminal carboxylic acid group in place of a methyl group.

**Figure 5.**
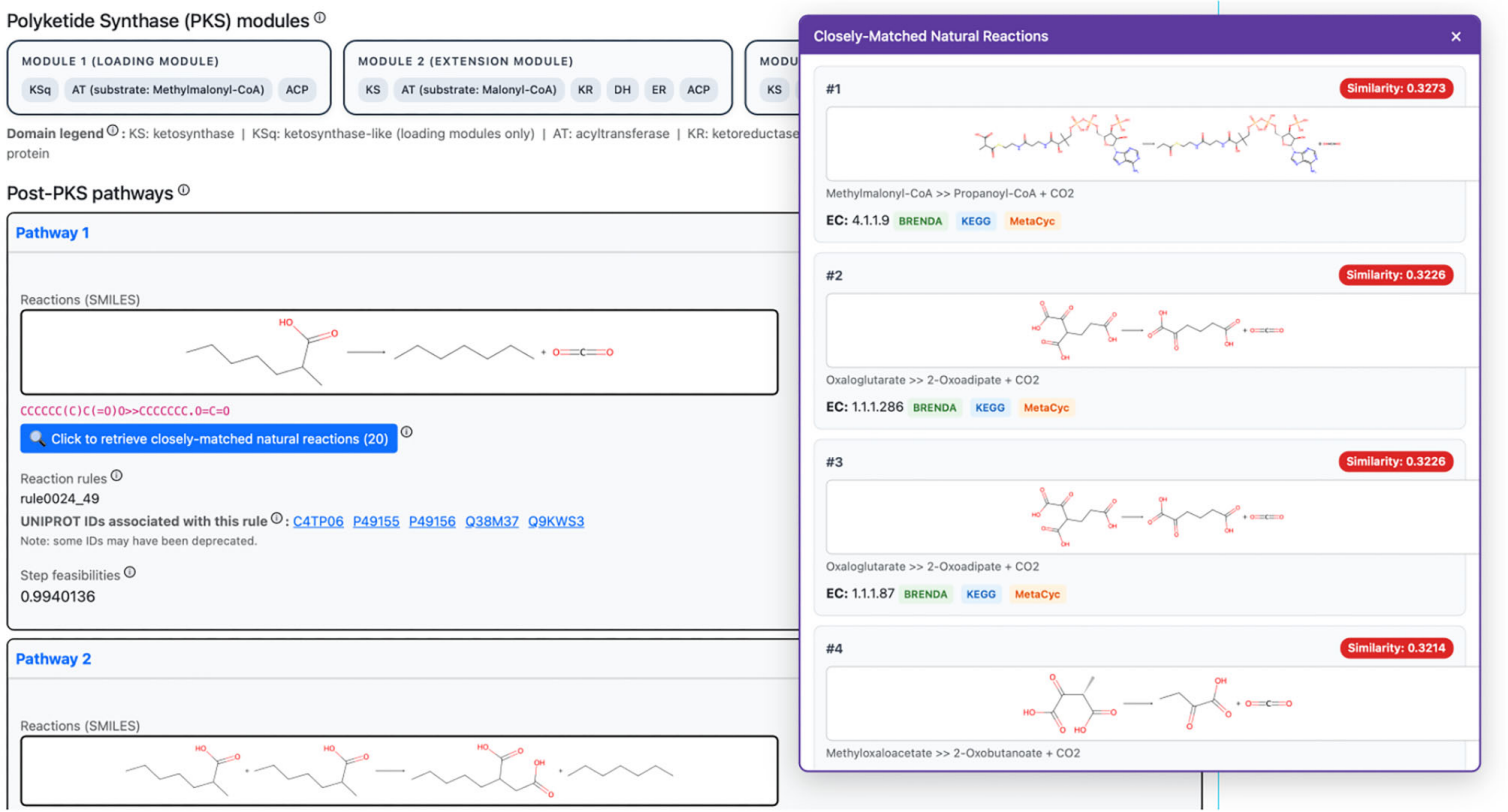
Enzyme and reported reaction retrieval for predicted biosynthetic reactions. For each enzymatic reaction step, users can retrieve closely matched natural reactions from a curated database of ∼55 000 known enzyme-catalyzed reactions. The floating panel (right) shows the top-ranked hits organized in descending order of reaction center Morgan fingerprint (RCMFP) Tanimoto similarity, along with EC classifications and links to BRENDA [61], KEGG [62–65], and MetaCyc [58]. Each reaction step also lists its associated DORAnet reaction rule, UniProt protein identifiers, and predicted step feasibility score.

### Retrieval of similar enzymes and reactions

To assist users in identifying candidate enzymes for each predicted enzymatic reaction, TridentSynth retrieves closely matched natural reactions from a curated database of 55 228 known enzyme-catalyzed reactions. This database was built by pooling fully atom-mapped reactions from versions of BRENDA [61], KEGG [62–65], and MetaCyc [58] released by the EnzymeMap database [66]. For each enzymatic reaction within a predicted pathway, TridentSynth computes a reaction center Morgan fingerprint (RCMFP) and searches for the most similar reactions within the same DORAnet rule family using the Tanimoto coefficient. Restricting the search to reactions that share the same rule family, i.e. the same generalized reaction center transformation ensures that retrieved hits are chemically relevant. Because enzymatic reactions may be catalogued in either direction, the search considers both orientations and reports the higher similarity score, while setting similarity to zero when reaction centers are not equivalent. The top 20 hits are returned for each query reaction and displayed in an interactive panel alongside their EC classification, source organism, and protein identifiers with links to BRENDA [61], KEGG [62–65], and MetaCyc [58], and UniProt [57]. This enables users to rapidly identify naturally occurring enzymes that catalyze transformations similar to those proposed by TridentSynth and to prioritize candidates for experimental validation. We note that this enzyme retrieval similarity score serves as a critical complement to the DORA-XGB reaction-level feasibility score described above. While the feasibility score assesses whether a proposed reaction is biochemically plausible at the reaction level, the enzyme retrieval score indicates how closely the proposed transformation matches known natural reactions at the enzyme level. Users should consider both scores together when deciding whether to proceed with experimental implementation, as neither score alone captures the full picture. For example, a reaction with a high feasibility score but a low enzyme retrieval similarity score may suggest that the transformation is chemically reasonable but lacks strong enzymatic precedent, and may therefore require enzyme engineering to realize experimentally.

### Input safety checks built into TridentSynth

TridentSynth incorporates multiple layers of input validation and safety screening to prevent the execution of invalid, unsupported, or inappropriate synthesis requests (Supplementary Fig. S3). Upon submission, all user-provided molecular inputs, whether entered as SMILES strings or drawn structures, are immediately parsed and validated using RDKit. Inputs that cannot be sanitized, neutralized, or interpreted as chemically valid molecules are rejected, and the user is presented with a clear error message prompting correction before any computation is initiated (Supplementary Fig. S3A). This early stage validation prevents failures arising from malformed chemical representations and wastage of compute resources.

In addition to structural validation, TridentSynth screens all submitted targets against a predefined list of controlled substances. If a user submits a compound found on this list, the system displays a warning notifying the user that the input may be a controlled or prohibited substance (Supplementary Fig. S3B). Users may still proceed with the synthesis if they have a legitimate research purpose. These safeguards are enforced prior to job submission to the computational backend, ensuring that users are informed before any restricted inputs are processed by RetroTide or DORAnet. Together, these safety checks provide a responsible user experience, discouraging misuse while maintaining accessibility for legitimate biosynthetic and cheminformatics research.

## Discussion

Here, we present TridentSynth (https://tridentsynth.lbl.gov), a freely available, web-based synthesis-planning platform designed to make integrated chemoenzymatic and PKS-based pathway design accessible to experimental scientists without requiring local installation or programming expertise. TridentSynth brings together multifunctional type I PKS-based biosynthesis, monofunctional enzymatic transformations, and synthetic organic chemistry reactions within a single interactive environment, allowing users to explore diverse synthesis routes through a unified and visually intuitive interface. By integrating these three complementary synthesis paradigms, TridentSynth substantially expands the chemical space accessible to computational retrosynthesis beyond what is achievable using chemistry or monofunctional enzymes alone.

Beyond pathway generation, our webtool places a strong emphasis on interpretability and responsible use. For pathways involving monofunctional enzymatic transformations, the platform provides machine-learning–derived feasibility scores that estimate the biochemical plausibility of individual reaction steps and, by extension, multistep pathways. At present, feasibility-based ranking is limited to monofunctional enzymatic reactions; PKS-based designs and synthetic chemistry reactions are presented without feasibility scoring but remain fully integrated within the broader pathway design framework. In addition, built-in validation and safety checks ensure robust handling of user inputs. When a controlled substance is detected, users are warned but may still proceed for legitimate research purposes, supporting the responsible deployment of our synthesis-planning software.

As a freely available webtool, TridentSynth is designed to encourage broad community adoption and extension. Its modular architecture enables researchers to suggest new features and improvements that are of interest to them on the platform. In future versions of the tool, we plan to also release an open source standalone desktop application version of TridentSynth, which users can download to run locally and offline. We anticipate that TridentSynth will serve not only as a practical synthesis-planning resource, but also as a community-driven foundation for continued methodological development in computational retrosynthesis and integrated chemoenzymatic pathway design.

## Supplementary Material

gkag471_Supplemental_File

## Data Availability

We have made TridentSynth free and open to all users and there is no login requirement. It can be accessed at https://tridentsynth.lbl.gov.
