# Supplementary material for: TridentSynth: a webtool for the retrosynthesis of molecules using chimeric type I polyketide synthases and chemoenzymatic pathways
Source: Nucleic Acids Res. 2026 May 11;54(W1):W65–76. doi: 10.1093/nar/gkag471 (PMC13355077; doi:10.1093/nar/gkag471)
Supplement: gkag471_Supplemental_File [file gkag471_supplemental_file.pdf]

Supplementary Information for

TridentSynth: A webtool for the retrosynthesis of molecules using chimeric type I polyketide synthases and chemoenzymatic pathways

Web architecture of the TridentSynth platform

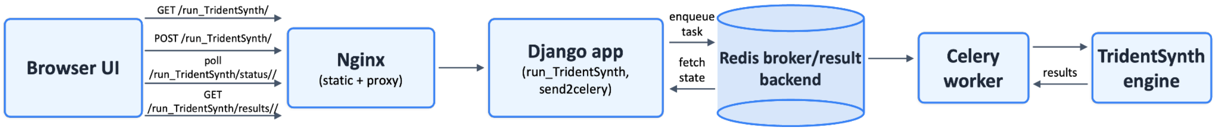

SI Figure 1. TridentSynthWeb system architecture. High-level architecture of the TridentSynthWeb platform, showing the browser interface, the Django backend (run_TridentSynth and send2celery apps), asynchronous execution via Celery with Redis as broker/result backend, and the TridentSynth engine. Nginx fronts HTTP traffic and serves static assets, while the results flow returns processed pathway designs to the user once computation completes.

Flow of requests and responses through the TridentSynth webtool

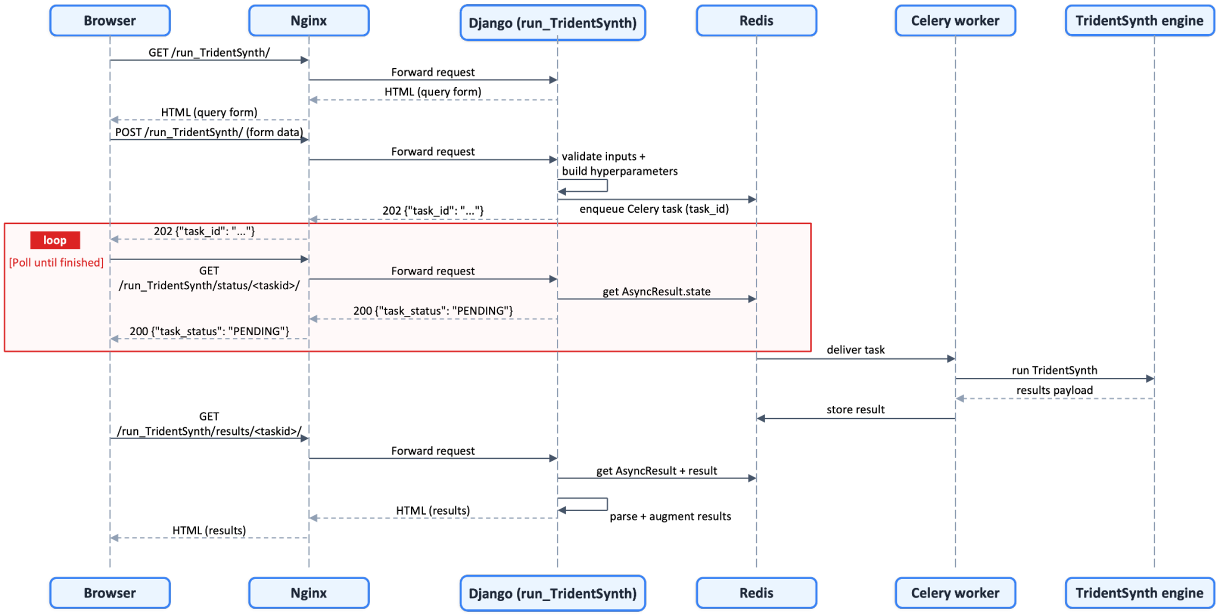

SI Figure 2. TridentSynthWeb request–response sequence. UML-style sequence diagram depicting a complete TridentSynth job lifecycle, from the user submitting a target SMILES in the browser, to Django validation and task submission, asynchronous execution by Celery with Redis state updates, polling for status, and final rendering of results back to the user.

Error handling and safety checks implemented by TridentSynth

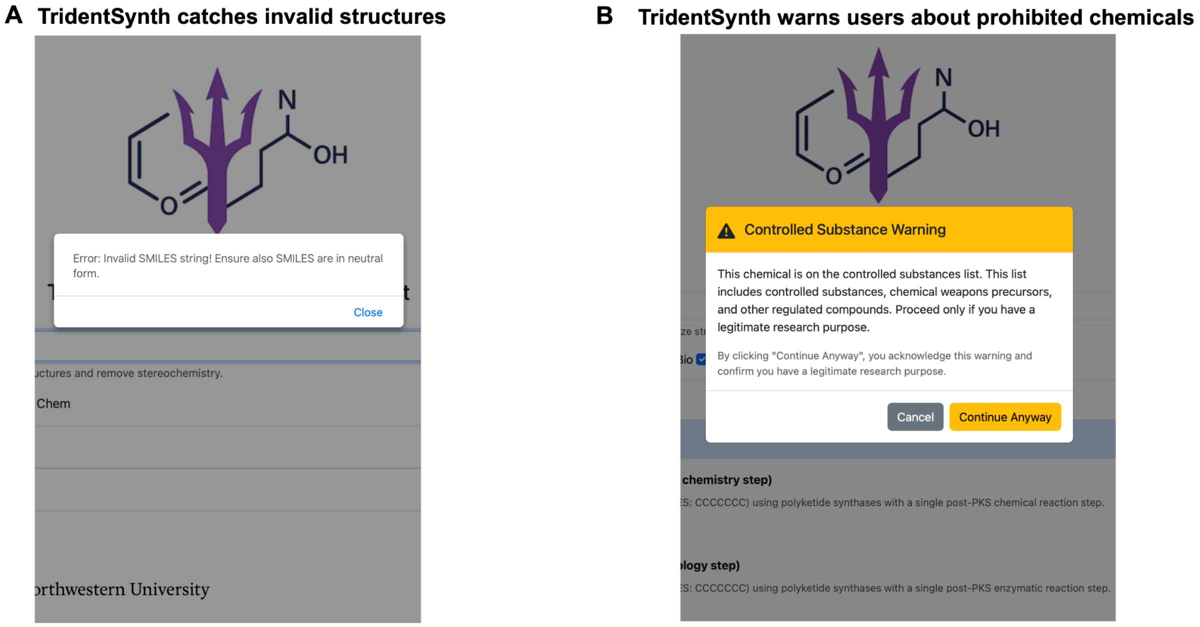

SI Figure 3. TridentSynth prevents users from entering invalid molecular structures or prohibited chemicals. **(A)** If a user enters a SMILES string or draws a molecular structure that cannot be processed by the popular cheminformatics software RDKit, TridentSynth prints an error and asks the user to check their structure again. **(B)** Additionally, if a user attempts to generate synthesis designs for a prohibited chemical, TridentSynth will warn the user before executing the requested synthesis.
